# BMDB: An integrated database and web platform for single-cell transcriptomic profiling of bone marrow microenvironment

**DOI:** 10.1016/j.csbj.2025.11.028

**Published:** 2025-11-15

**Authors:** Jialin Chen, Hao Yu, Chunjing Bian, Yifei Hu, Ke Sui, Xi Zhang, Zheng Wang

**Affiliations:** a Jinfeng Laboratory, Chongqing 401329, China; b Medical Center of Hematology, Xinqiao Hospital, State Key Laboratory of Trauma, Burn and Combined Injury, Army Medical University, Chongqing 400037, China; c Department of General Surgery, Xuanwu Hospital, Capital Medical University, Beijing 100053, China; d Department of Biomedical Engineering, The University of Texas Southwestern Medical Center, Dallas, TX 75390, USA; e Bio-Med Informatics Research Center & Clinical Research Center, The Second Affiliated Hospital, Army Medical University, Chongqing 400037, China

**Keywords:** Bone marrow niche, Single-cell RNA sequencing, Web server, Reference atlas

## Abstract

The bone marrow microenvironment orchestrates hematopoiesis and disease processes, yet its profound cellular and molecular complexity remains incompletely resolved. While single-cell RNA sequencing offers unprecedented resolution, the lack of a unified resource integrating niche-specific data across health and disease states, and developmental stages hinders systematic exploration. Here, we present BMDB (https://bmdb.jflab.ac.cn:8084/), an integrated database and web platform aggregating 435,682 single cells from 142 healthy and 82 pathological human and mouse samples, establishing species- and stage-specific reference atlases that harmonize niche cell annotation, improve cross-study comparability, and enable translational analyses. Its interactive web platform provides modules for functional exploration, reference-guided analysis, dynamic comparison across states, and knowledge graphs linking genes, variants, pathways, and pathologies. Additionally, users can upload their own scRNA-seq datasets to map onto the reference atlas for cell type annotation and downstream analyses. Collectively, BMDB provides high-quality reference atlases and interactive tools, serving as a valuable resource for investigating bone marrow microenvironment heterogeneity and hematopoietic regulation.

## Highlights

• BMDB is the first dedicated single-cell transcriptomic resource of the bone marrow niche, incorporating 435,682 single cells from 142 healthy and 82 pathological human and mouse samples.
• BMDB serve as a user-friendly web platform, performing one-click analyses, and uploading users’ datasets for annotation and downstream analysis using the integrated reference atlas.
• BMDB has constructed three high-quality single-cell transcriptomic atlases of the bone marrow niche, with comprehensive annotations of cell types and lineages.
• BMDB integrates a knowledge graph linking genes, variants, pathways, and pathologies, facilitating efficient exploration of molecular mechanisms in bone marrow microenvironment.

## Graphical Abstract

**Figure:**
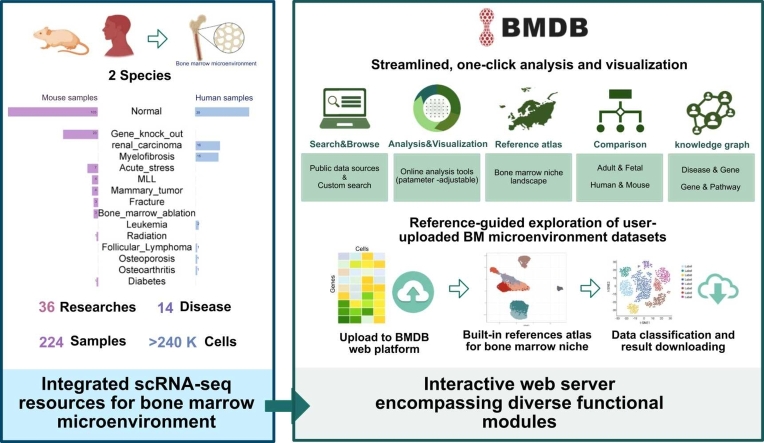

## 1 Introduction

Hematopoiesis is the process by which all mature blood cell types arise from hematopoietic stem and progenitor cells (HSPCs), and this occurs within the specialized bone marrow (BM) niche that orchestrates stem cell maintenance, differentiation, and trafficking. Interplay between HSPCs and stromal, endothelial, osteolineage, immune, and other niche components tightly regulates hematopoiesis. Under homeostatic conditions, these cellular and molecular crosstalk networks maintain balanced blood production, with signals from bone marrow (BM) niche dynamically adjusted to physiological demand [1]. However, emerging evidence indicates that hematological malignancies profoundly reshape niche architecture and function [2], [3]. For example, in multiple myeloma, malignant plasma cells reprogram MSCs, endothelial cells, and immune subsets to create a pro-inflammatory, immunosuppressive microenvironment that supports tumor survival and drug resistance [4]. In acute leukemias, leukemic blasts remodel stromal and vascular compartments, generating aberrant cytokine milieus and adhesion interactions that inhibit normal hematopoiesis while favoring malignant expansion [5], [6]. Beyond malignancy-driven remodeling, the BM niche status is also critical for the success of hematopoietic stem cell transplantation (HSCT). Pre-transplant conditioning regimens, including high-dose chemotherapy and irradiation, induce acute and long-term damage to stromal and vascular compartments, impairing donor HSC homing, lodging, and reconstitution [7]. Strategies such as MSC co-transplantation and other niche-targeted interventions have shown promise in preclinical and clinical studies by ameliorating niche injury, enhancing engraftment, and reducing complications [8], [9]. Despite these advances, the inherent complexity, cellular heterogeneity, and dynamic remodeling of both healthy and diseased BM niches remain incompletely understood.

In recent years, the advancement of single-cell RNA sequencing (scRNA-seq) technology has presented an unparalleled chance to investigate the intricate cellular diversity and complex microenvironment linked to various developmental, physiological, and pathophysiological processes [10], [11], [12]. These processes include hematopoiesis, inflammation, neurodegenerative diseases, and cancer [13], [14]. Notably, in the field of cancer research, there has been a significant surge in both the quantity and quality of scRNA-seq data over the past few years [15], [16]. The extensive amount of public scRNA-Seq data necessitates the implementation of efficient quality control measures, platform and analysis evaluation, data mining and integration, and information management. Consequently, databases like CancerSCEM and TISCH have been established to curate the accumulating data and offer single-cell gene expression profiles in the field of cancer biology [17], [18]. In comparison to solid tumor tissue, hematopoietic tissue, such as bone marrow, presents a more convenient option for scRNA-seq investigations due to its accessibility as a single-cell suspension. Furthermore, the presence of established molecular markers and functionally validated cell types adds to the appeal of studying the hematopoietic system [19], [20], [21]. Recent significant studies have shed light on the molecular intricacies of the BM niche in mice, leading to a revised understanding [22], [23]. For example, Baryawno et al. have identified 17 distinct cell types with varied functions in the murine BM stroma, emphasizing its complexity [24]. In recent years, a number of tissue-centric single-cell databases have been developed, advancing our understanding of organ-specific cell diversity. However, there is currently no available database to effectively utilize the abundant scRNA-seq data resources pertaining to the dynamic BM niche, especially for comprehensively analyzing the heterogeneity of BM microenvironment and dissecting the hematopoiesis mechanisms (Supplementary Table 1).

To bridge this critical gap, we developed BMDB, a web-based portal that systematically aggregates, processes, and visualizes bone marrow scRNA-seq data across human and mouse, healthy and diseased samples. BMDB uniquely consolidates and re-analyzes 435,682 single cells from 224 human and mouse samples, spanning healthy and diseased states into species- and stage-specific reference atlases, enabling standardized cross-dataset annotation. Its four interactive modules including “exploration”, “reference”, “comparison” and “knowledge” provide an unprecedented suite for mechanistic discovery. Crucially, BMDB’s de novo analysis capability allows users to map their own data onto these validated atlases, transforming fragmented data into a unified, community-driven resource. By providing a unified, interactive framework for mapping new datasets onto reference atlases, interrogating niche heterogeneity, and exploring mechanistic relationships via knowledge graphs, BMDB constitutes an original and powerful resource poised to accelerate insights into bone marrow microenvironment biology and to inform therapeutic strategies.

## 2 Materials and methods

### 2.1 Data collection

We searched PubMed and Google scholar for the literature related to single-cell RNA-seq and bone marrow niche using the following keywords: ‘((‘single-cell RNA’) OR (scRNA-seq)) AND (bone marrow niche OR bone marrow microenvironment). All datasets were collected before January 2025. We then manually curated the publications using three criteria: 1) the data were publicly available; 2) the samples must be derived from bone marrow with a classical non-hematopoietic FACS panel (e.g., LEPR⁺/CD45⁻/CD117⁻/Ter119⁻); and 3) the cell type annotations from publications must include bone marrow non-hematopoietic cells.

Finally, a total of 36 bone marrow niche-related datasets were obtained from this systemic search, which comprise 142 datasets of normal samples and 82 of pathological samples. By manually reviewing each publication and its supplements, we curated metadata of each dataset—species, disease state, developmental stage, FACS gates, and sequencing method.

### 2.2 Data processing pipelines

*Datasets preprocessing* For further processing and analysis, high-quality data from each dataset were meticulously chosen. Seurat [25] (v4.3.0) was employed to generate a unified format. Considering the methodological differences among sequencing platforms, quality control thresholds were determined based on both the original publications and commonly adopted criteria in the field. For droplet-based technologies, cells with < 500 transcripts, > 10 % mitochondrial content, or > 5000 detected genes were excluded, and genes expressed in fewer than three cells were removed. For the plate-based dataset (GSE128743), cells with fewer than 1000 detected genes or greater than 10 % mitochondrial reads were filtered out, while genes expressed in fewer than three cells were also discarded. In all datasets, cells expressing predefined hematopoietic markers (CD3A, CD3B, CD3C, CD3D, CD3E, CD4, CD8A, CD8B, CD8E, CD8G, CD19, TFRC, LY6G, LY76, VWF, PTPRC, ITGAM) were excluded. The filtered data were normalized with LogNormalize, and highly variable genes (top 3000, VST method) were identified for PCA. The first 10 principal components were used to build a k-nearest neighbor graph (k = 20), followed by shared nearest neighbor clustering using the Jaccard index and Louvain algorithm (resolution = 2.0). Dimensionality reduction was performed with UMAP (cosine metric, uwot v0.1.14).

*Cell type annotation* Each dataset that has undergone standardized preprocessing was uniformly re-annotated for cell type classification. The unified annotation was independently transferred from the reference atlases we built for mouse and human using scvi-tools [26] (v1.0.3). We employed scANVI, a semi-supervised deep learning model implemented in the scvi-tools framework. Specifically, we took the raw counts without any normalization as input to scVI and selected the aforementioned 3000 HVGs as the input features. Then we trained reference dataset using the standard scVI workflow, setting the "gene_likelihood = 'normal'" and training the model with "max_epochs = 200", while keeping all other parameters at their default values. Subsequently, the pre-trained scVI model was extended with scANVI with default parameters. Finally, we mapped the sample data to the reference and assigned cell type labels based on the pre-annotated reference atlas.

### 2.3 Advanced data processing

*Trajectory analysis* To conduct the analysis of developmental trajectories, we employed monocle2 [27] (v2.32.0) algorithms to establish two distinct analytical frameworks. Subsequently, lineages comprising more than 50 cells were selected for subsequent investigation. The examination of developmental trajectories encompassed all identified lineages. Within each lineage, the genes expressed in a minimum of three cells, as well as the HVGs, were identified and considered as ordering genes. To reduce dimensionality, the DDRTree algorithm was employed.

*Cell-cell communication* We applied CellChat [28] (v2.1.2) to predict intercellular communication within each tissue, based on receptor–ligand interactions. For every two cell types, the mean co-expression of receptor–ligand pairs was calculated. Cell type labels were then randomly permuted to disrupt biological associations, generating null distributions specific to each interaction. Interaction pairs with co-expression values that were found to be significantly higher than the background (P < 0.05) were selected and subsequently included in the BMDB, along with receptor-ligand profiles for each cell type in all tissues. These significant interactions, together with receptor–ligand profiles across tissues, were incorporated into BMDB to facilitate systematic exploration of communication networks.

*Regulatory network inference* PySCENIC [29] (v0.12.1) was employed to infer the gene regulatory network in BMDB datasets with default settings. It applied GENIE3, cisTarget, and AUcell algorithms to identify transcription factors and their targeted genes. The regulon specificity scores were calculated by the calcRSS function to access regulon activities.

### 2.4 Construction of single-cell BM niche reference atlas

The construction of the three comprehensive atlased of the bone marrow niche was based on three representative datasets [24], [30], [31]. Following data collection, the construction of a reference atlas was carried out using a "five-step" strategy: 1) quality control of the scRNA data from each individual sample, 2) normalizing, 3) dimensionality reduction, 4)cell clustering, and 5) annotating cell types in the integrated data. The preprocessing of the reference datasets followed the same procedure as described above for droplet-based data.

In the second phase of our methodology, filtered data were normalized using *LogNormalize*, scaled to [0,10], and integrated across samples using 3000 features identified by *SelectIntegrationFeatures*. Each dataset was scaled and processed by PCA, and anchors were identified via RPCA for robust integration [25]. After integration, 3000 HVGs (vst method) were selected for PCA, with the top 10 PCs (ranked by standard deviation) retained for UMAP dimensionality reduction. UMAP was run with min.dist = 0.05 for high-resolution visualization. Clustering was performed using the top 10 PCs to construct a kNN graph, with Jaccard index–based sNN graphs and the Louvain algorithm (resolution = 0.8) applied to identify clusters.

Cell type annotation relied on canonical markers, assigning major BM niche cell populations: mesenchymal stromal cells (MSCs), osteolineage cells (OLCs), fibroblasts, endothelial cells (ECs), chondrocyte-like cells (CLCs), and pericytes. Subclusters were further defined by identifying cluster-specific marker genes using FindMarkers, followed by lineage trajectory analysis. DDRTree was applied to Louvain subclusters, and those with overlapping trajectories were merged. Differential expression analysis between subclusters identified unique markers, enabling annotation of refined niche subtypes.

To ensure consistency across datasets, BMDB also incorporates a Cell Ontology–based standardized annotation system. Following SSSOM and SEMAPV guidelines, both labels were mapped to the Cell Ontology (CL) term graph using lexical matching, followed by manual curation [32]. Exact correspondences were recorded as “skos:exactMatch,” while broader mappings to parent terms were noted as “skos:broadMatch.” Species and tissue of origin are also captured in standardized fields.

A comparable approach was employed to create the atlas of the human BM niche from both developmental stages, albeit with minor adjustments. The marker genes utilized for annotating the primary cell types or subclusters were sourced from published literature.

### 2.5 Comparison across stages

Based on the previously described datasets that constructed human fetal and adult reference atlas [30], [31], we conducted a comparative analysis between developmental stages. Briefly, non-hematopoietic cells were extracted and integrated into one unified dataset, with batch effects removed using the RPCA method with default parameters. Subsequently, following the previously described steps, the integrated data underwent dimensionality reduction and clustering while preserving the original cell type classifications from the reference atlas. The clustering resolution was manually adjusted until each cell type was consolidated into a distinct cluster. Finally, we performed downstream analyses, including differential gene expression analysis, pseudotime trajectory analysis using monocle2, and gene regulatory network inferring using pySCENIC.

### 2.6 Knowledge graph generation

We utilize Neo4j (v4) (https://neo4j.com/) as the graph database engine to integrate the complete disease network from Clinical Knowledge Graph [33] (CKG) and construct a comprehensive knowledge graph related to genes and proteins. BMDB offers two knowledge-matching modes, enabling users to incorporate gene-related analyses. The first mode identifies mutations associated with both proteins and diseases, along with relevant research findings. The second mode maps pathways linked to specific proteins and their metabolic products.

### 2.7 Quantitative benchmarking across annotation algorithms

We compared the mapping approaches implemented in BMDB(scANVI and RPCA) with three widely used tools: CellTypist, SingleR, and scmap. Independent bone marrow niche datasets from both mouse(GSE249528) and adult human(GSE241825) samples were employed as test data [34], [35]. For each method, the predicted cell-type labels were evaluated against the manually curated annotations from the original publications. Quantitative metrics including overall accuracy, macro F1-score, micro F1-score, weighted F1-score, and area under the receiver operating characteristic curve (AUROC) were computed using the scikit-learn library (v1.3.2).

### 2.8 Out-of-domain generalization and rejection testing

To assess model generalization, independent PBMC and lung single-cell datasets were used as out-of-domain (OOD) controls, while the bone marrow niche dataset served as the in-domain (ID) reference. For each cell, confidence scores were derived from the prediction probability matrices generated by BMDB integrated mapping models. The distributions of confidence scores were visualized using violin and box plots to summarize mean and median values across datasets. In addition, coverage–threshold and accuracy–threshold curves were plotted to evaluate the effect of different confidence thresholds on cell retention and annotation accuracy.

## 3 Results

### 3.1 Contents and framework of BMDB web server

**Fig. 1:**
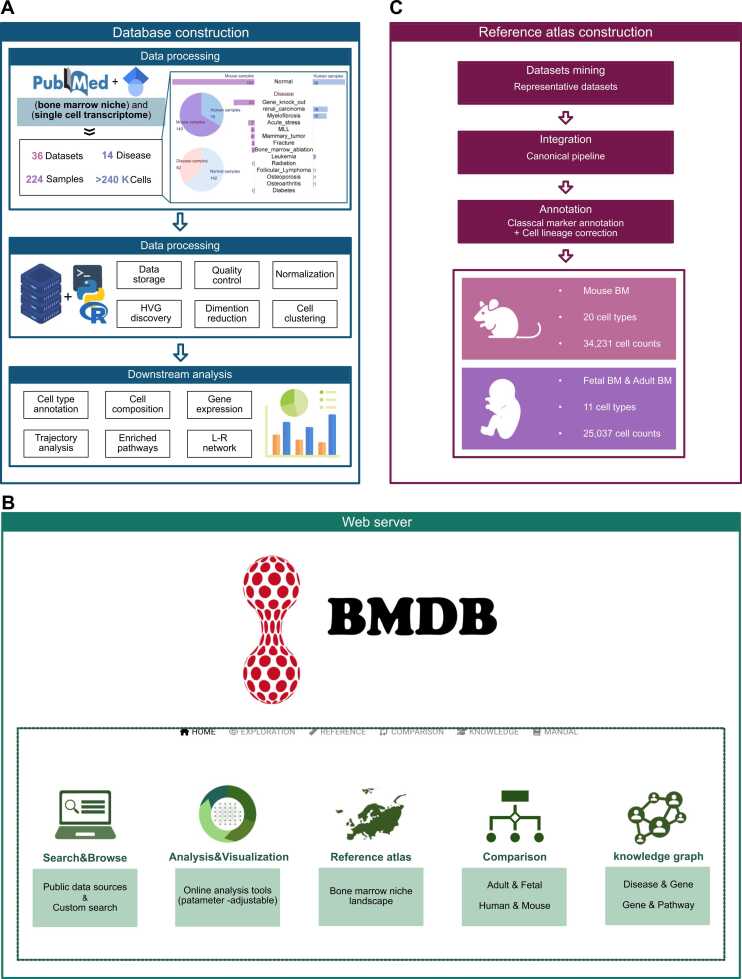
Scheme of BMDB database and web server pipeline. (A) Data collection, data statistics, data preprocessing and downstream analysis pipeline of BMDB. (B) The integrated functional modules in BMDB web server. The website features analysis and visualization of built-in datasets and reference atlases, as well as the online analysis for user’s uploaded data. (C) Workflow of constructing reference atlas of BM niche. High-quality single-cell reference atlas of mouse and human BM niche based on the representative datasets.

The current version of BMDB (https://bmdb.jflab.ac.cn:8084/) was established from 36 bone marrow niche-related scRNA-seq researches in Homo sapiens and Mus musculus, involving 435, 682 single cells (Fig. 1A**,** Supplementary Table 2). Among them, 142 samples were generated from normal tissues and 82 samples were generated from pathological tissues of 14 kinds of disease (Fig. 1A, Supplementary Table 2). The framework of BMDB web server encompasses four primary functional groups: "Exploration" for data management and for display of integrated datasets, "Reference" for investigating reference atlases and for users' data annotation, "Comparison" for exploring the developmental differences between adult and fetal groups, and "Knowledge" for enhancing imformation integration, mining, and discovery (Fig. 1B, Fig. 1C).

### 3.2 Features and utilities

**Fig. 2:**
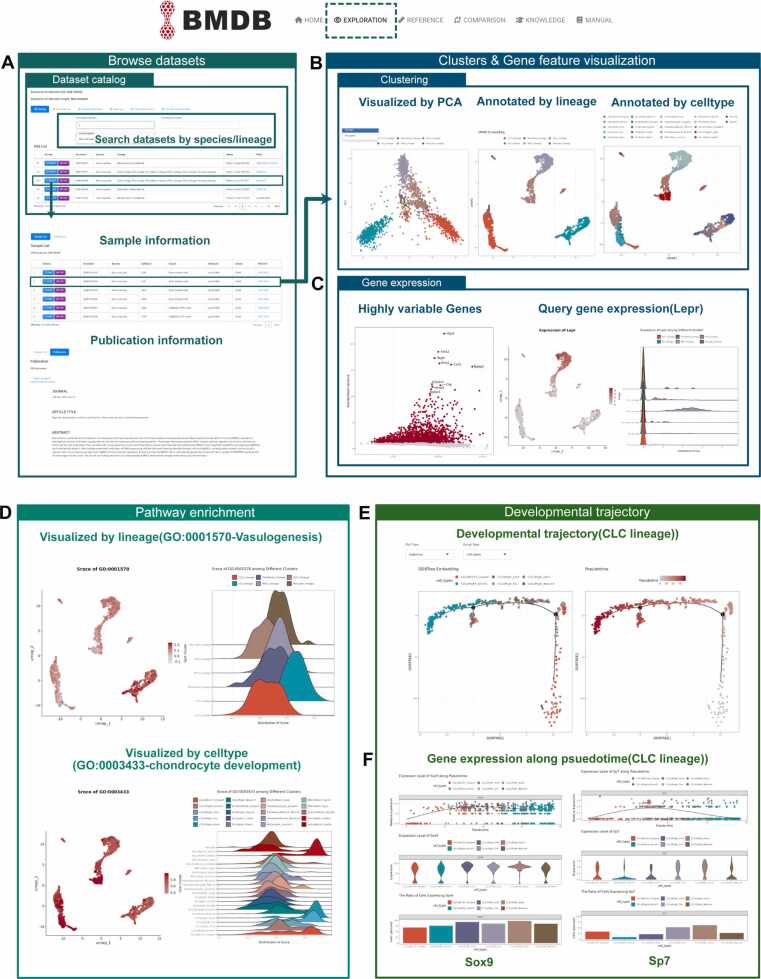
Primary display of built-in datasets analysis in BMDB. (A) The present study showcases the effective management and querying of datasets in the BMDB. The dataset catalog, organized based on articles, enables users to retrieve specific samples and the publication information. (B) Within "Data Overview" module, cell type and lineage type annotation are presented using UMAP and PCA plot. (C) Interface of browsing gene expression pattern across cell types. The expression of selected gene (*Lepr*) in different cell types can be shown by UMAP or by violin plots. (D) In "Pathway Enrichment" module, UMAP plot visualized the expression of selected GO-enriched pathways, while a river plot illustrates their differences across cell types or lineages. (E) DDRTree showed the pseudotime trajectory of CLCs lineage. (F) Scatter plot showed selected gene expression dynamics along the pseudo-temporal developmental trajectory. Violin plot and bar plot depicted the gene expression level across subclusters within the selected lineage.

*Data browsing and basic display* The dataset management process was conducted by BMDB utilizing an internally developed pipeline. Within the "Exploration" group, users were enabled to search datasets based on species, and cell lineages. The detailed information for each dataset is listed on the webpage, including the GSE accession, species, cell lineage, and publication information. Upon selecting a dataset, the specific information for each sample is displayed in the "Sample List" page, comprising the secondary accecion, cell count, cell sorting imformation, sequencing platform and library details, while the "Publication" page displays the publication date, title, and abstract of the article (Fig. 2A).

By clicking the "LOAD" button of the interested sample, the "Data Overview" page reveals the cell clustering, gene expression and quality control results of selected dataset. Users can have the option to select from different dimensionality reduction and cell annotation methods. The platform provides PCA and UMAP for dimensionality reduction and visualization, while cell annotation options include cell type classification and lineage annotation (Fig. 2B). Furthermore, users could highlight the interested cell types or lineages based on their research needs utilizing PCA or UMAP plots. Users can easily explore gene functions using BMDB, which not only intuitively presents highly variable genes (HVGs) within samples but also provides approaches for comparative analysis across different cell types of lineages for genes of interest. For example, by choosing a gene (Lepr) from the provided gene list, the expression pattern of the selected gene was demonstrated using the tabular format and UMAP plot, while its differential expression across various cell lineages was presented using the ridge plot (Fig. 2C).

*Extended display of built-in data* In addition, the "Exploration" functional module allows users to conduct various complex analyses, such as pathway enrichment cell differentiation trajectory, gene regulatory network, and cell-cell communication. The "Pathway Enrichment" module facilitates the execution of pathway analysis on cell clusters. Through the selection of specific pathways, such as GO:0001570 ("Vasculogenesis"), and GO:0003433 ("chondrocyte development"), from the provided GO terms format, users are able to represent their expression patterns using UMAP plots and compare their differentially expression across various cell types or cell lineages using ridge plots. For instance, the expression of pathway GO: 0001570 is predominantly observed in EC lineages, whereas the expression of pathway GO: 0003433 is primarily expressed in OLCs and CLCs cell types, with the most pronounced expression found in the OLCs/Ndnf-_Col8a1- subgroup (Fig. 2D).

On the "Trajectory" page, users have the option to customize several parameters in order to obtain the temporal-spatial development patterns of the chosen cell lineage through a drop-down box (Fig. 2E). By selecting via the "Group Type" drop-down box, pseudotime trajectory could be visualized by cell subtypes or state caculated by monocle2. To investigate the involvement of the gene of interest in the development of a specific cell type, users can visualized the choosen gene expression dynamics along the pseudotime trajectory by cell subtypes or state. As shown in the visualization of Sox9 and Sp7 expression trends along pseudotime and their expression levels across subclusters, Sox9 exhibits sustained expression throughout the trajectory, whereas Sp7 shows a decline in expression over pseudotime (Fig. 2F).

**Fig. 3:**
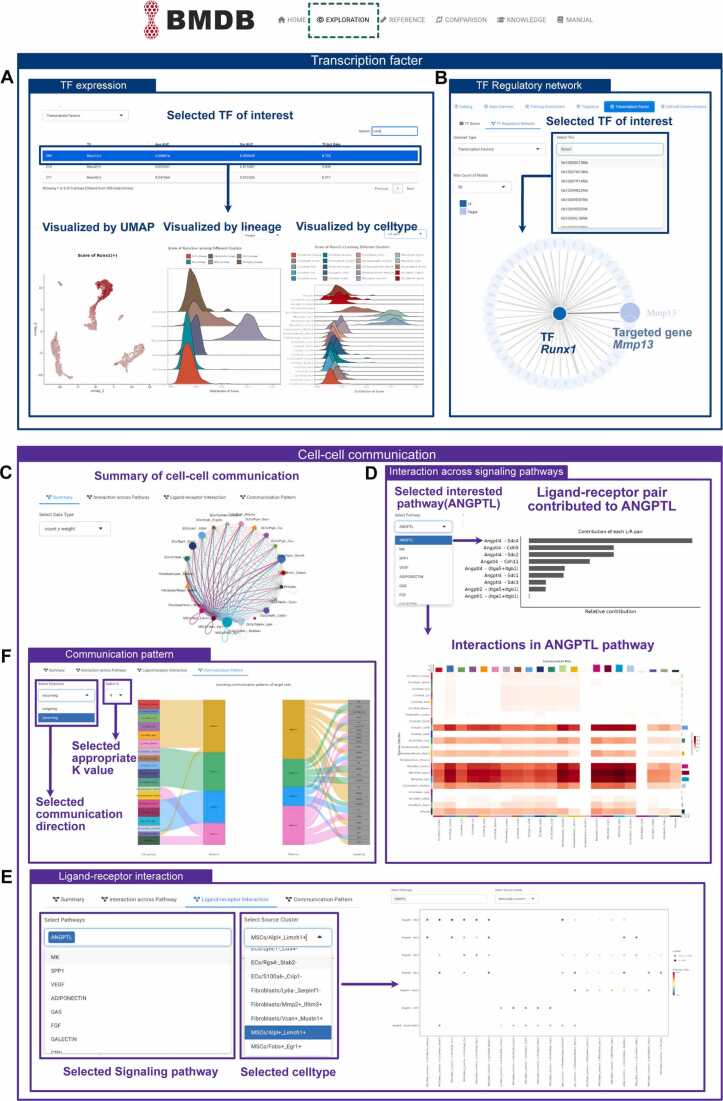
Extended display of built-in data analysis in BMDB. (A) Within "Transcription Factor" module, UMAP plot and violin plot showed the expression level of selected transcription factor. (B) Network diagram visualized the transcription factor *Runx1* and its top 50 specifically regulated target genes. (C) In the "Cell-cell Communication" module, circle plot illustrated the total interaction strength between any two cell types. (D) Bar plot and heatmap visualized contribution of each ligand-receptor pair to the ANGPTL signaling pathway. (E) Bubble plot showed the interactions between MSCs/Alpl+ _Limch1 + and other cell types within ANGPTL signaling pathway. (F) River plot visualized the incoming patterns between the cell groups and signaling pathway patterns.

Within the "Transcription Factor" module, users can investigate the expression patterns of selected transcriptional factor and their corresponding regulatory gene networks (Fig. 3A). On the "TF Score" page, a table lists transcription factors along with their AUC scores. Upon selecting a transcription factor of interest, users can visualize its expression levels through the UMAP plot, while a ridge plot displays its expression variations across cell types or lineages. As observed, Runx1 is predominantly expressed within the MSCs lineage (Fig. 3A). Besides, this module allows the exploration of targeted genes of interested transcription factors. For example, by selecting the Runx1 from the dropdown menu and setting the maximum number of displayed nodes to 50 generated the network diagram displaying Runx1 alongside its top 50 associated targeted genes, such as Mmp13 (Fig. 3B).

The module "Cell-cell Communication" provides users with the ability to analyze the process of cell-cell communication. There are four aspects of ligand-receptor analysis in this module, the overall intercellular communication strength, the expression levels of ligand-receptor pairs across all cell types, the ligand-receptor pairs expression within selected cell types, and the inferred global signaling patterns. The circle plot summarized the total interaction strength across all cell types of selected dataset (Fig. 3C). Utilizing the ANGPTL signaling pathway as an example, we illustrated the interactions within the ligand-receptor signaling network explicitly. On the "Interaction cross Pathway" page, the bar plot displayed the contribution of each ligand-receptor pair to the overall ANGPTL signaling pathway. The impact of ANGPTL signaling pathway across cell types could be visualized by heatmap, chord and circle plot. This heatmap showed that the ANGPTL signaling pathway primarily mediates communications within MSCs subgroups (Fig. 3D). Users can explore the strength of ligand-receptor pairs between a selected cell type and other cell types in "Ligand-receptor Interaction" page. The bubble plot depicted the expression level of ligand-receptor pairs constituting the ANGPTL signaling pathway between the cell pairs associated with MSCs/Alpl+ _Limch1 + subcluster. Angptl4-sdc4 exhibits a significant role in facilitating communication between MSCs subcluster and CLCs subcluster (Fig. 3E). Finally, we inferred the communication patterns within the datasets. By selecting an appropriate K value, the incoming signals of different cell types were classified into four distinct patterns. The river plot intuitively illustrated the associations between communication patterns, cell groups, and signaling pathways. Notably, we observed substantial differences in incoming signal patterns across different lineages (Fig. 3F).

*Reference atlas* To standardize and streamline cell type annotation for the bone marrow microenvironment, we provided the unified reference for cell type annotation separately for human and mouse datasets within BMDB. The construction of three high-quality atlases for mouse bone marrow niche cells, human bone marrow niches from both adult and fetal stages were accomplished by utilizing a pipeline that integrates canonical analysis methods [24], [30], [31] (Fig. 1C, Supplementary Figure 1A and Supplementary Figure 1B). For the human references, we integrated two representative datasets of bone marrow niches corresponding to different developmental stages, comprising a total of 21 samples, and generated stage-specific reference atlases accordingly. The cell type annotation of references were determined through the two-step process. Firstly, the original cell type annotations from the source publication are considered, with hematopoietic cells excluded. Subsequently, a final manual correction of cell types based on the expression patterns of canonical marker genes was executed to establish the comprehensive transcriptome references **(**Supplementary Figure 2**,** Supplementary Figure 3**,** Supplementary Table 3**)**. For the mouse reference, we integrated 11 samples from two bone marrow datasets to generate the reference atlas. The annotation of integrated reference categorized the cells into six lineages, namely MSCs, Chondrocyte-like cells(CLCs), Osteolineage cells(OLCs), fibroblasts, Endothelial cells(ECs), and pericytes. Further refinement of the annotation was carried out for each cell type, employing marker genes identified by FindMarkers (Supplementary Figure 4**,** Supplementary Table 3).

**Fig. 4:**
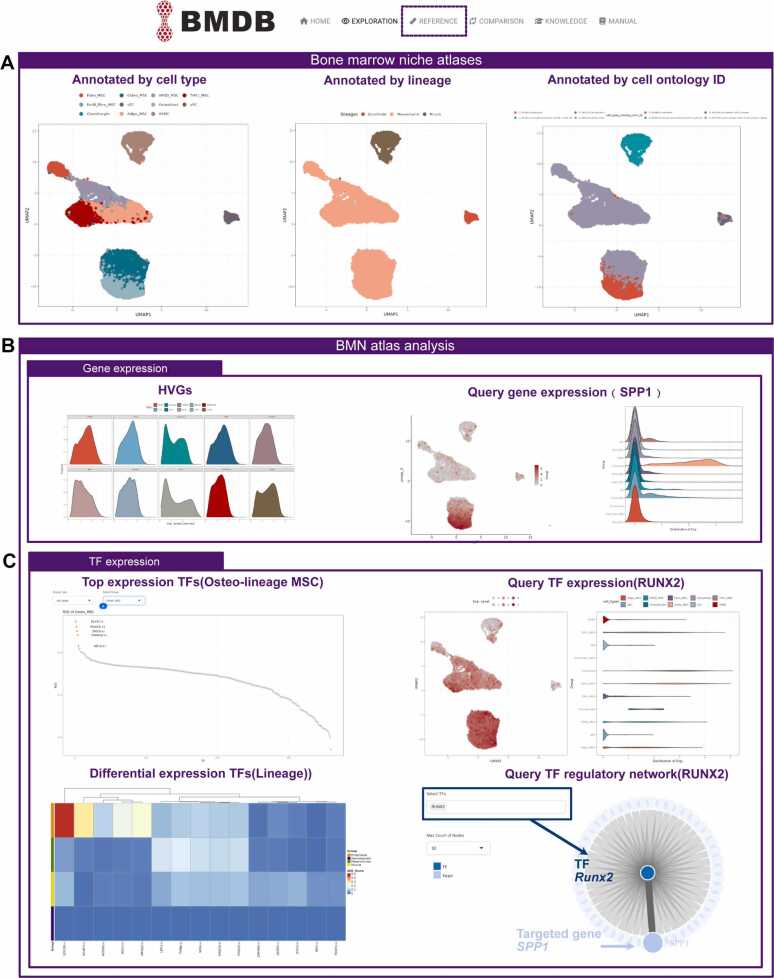
Reference atlases in BMDB. (A) Within "Reference" module, UMAP plots presented cell type, cell lineage, and standardized Cell Ontology ID annotations for the human adult bone marrow microenvironment. (B) The expression patterns of highly variable genes and queried genes within the human BMN reference atlas in the adult stage. (C) The expression patterns of activated transcription factors within the human BMN reference atlas in the adult stage.

The "Reference" module listed all the processed datasets and associated information. For further analysis and visualization, users can view the UMAP of integrated atlas and detailed demonstration of cell classification and genes expression patterns. BMDB provides three complementary levels of cell annotation, including fine-grained cell types, well-defined lineage categories, and standardized Cell Ontology labels. (Fig. 4A, Fig. 4B, Supplementary Table 4). BMDB enabled users to conduct an in-depth exploration of transcription factors within the reference atlas. On the "Transcription Factor" page, users could identify transcription factors that exhibit the highest specificity within a specific cell type or lineage, as well as the differentially expression of transcription factors across cell types or lineages. Additionally, users can investigate the expression profiles of transcription factors of interest across various cell types or lineages and analyze their corresponding gene regulatory networks (Fig. 4C).

**Fig. 5:**
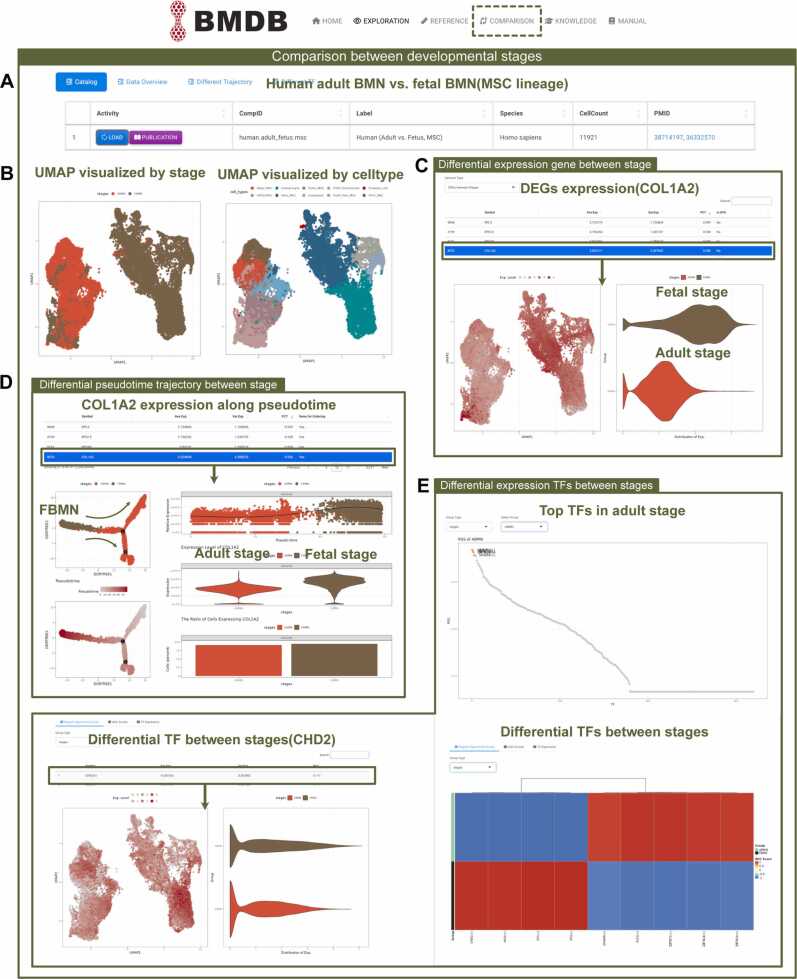
Comparative analysis across developmental stages in BMDB. (A) Within "Comparison" module, diagram showed the basic information of integration data of human BMN atlases across fetal and adult stages. (B) UMAP plots presented cell type annotations and stages of human bone marrow microenvironment across two developmental stages. (C) UMAP plot and violin plot showed the differential expression of the queried gene across different developmental stages. (D) DDRTree showed the pseudotime trajectory of human BMN atlases across fetal and adult stages. Violin plot illustrated the selected gene expression along peudotime trajectory. (E) The expression patterns of differential transcription factors within the human BMN reference atlas across developmental stages.

*Comparisons between adult and fetal stage* To investigate the phenotypic landscapes and interactions between different developmental stages, we integrated and re-analyszed human bone marrow niches from both fetal and adult stages (Fig. 5A). Within the "Comparison" module, UMAP plot was employed to visualize the cellular composition of the human bone marrow microenvironment across different developmental stages, highlighting significant differences between stages. The abundance of chondrocytes and proliferative chondrocytes significantly declines from the fetal to the adult stage. As for the MSCs lineage, fibroblast-related stem cells ("Fibro_MSC") predominate during the embryonic stage, whereas adipose-associated stem cells ("Fibro_MSC") are more prevalent in the adult stage (Fig. 5B). BMDB also enabled the querying and visualization of differentially expressed genes across various developmental stages (Fig. 5C). Additionally, users could easily perform pseudotime analysis across different developmental stages, facilitating the study of genes that change dynamically during bone marrow development. For example, we observed a gradual decrease in COL1A2 expression during development progresses (Fig. 5D). Finally, users can identify transcription factors that are specifically expressed at a particular developmental stage, as well as those differentially expressed across two developmental stages, with multiple visualization options available (Fig. 5E).

*Knowledge graph of diseases* To comprehensively integrate existing domain knowledge, facilitate efficient queries on the relationships between specific biomolecules or variants and relevant diseases, and identify novel disease-associated genes, mutations, or pathways, BMDB web tool incorporates a knowledge graph function named "Knowledge" module. BMDB includes two matching modes: (1) Gene/Protein to Disease and (2) Gene/Protein to Pathway.

**Fig. 6:**
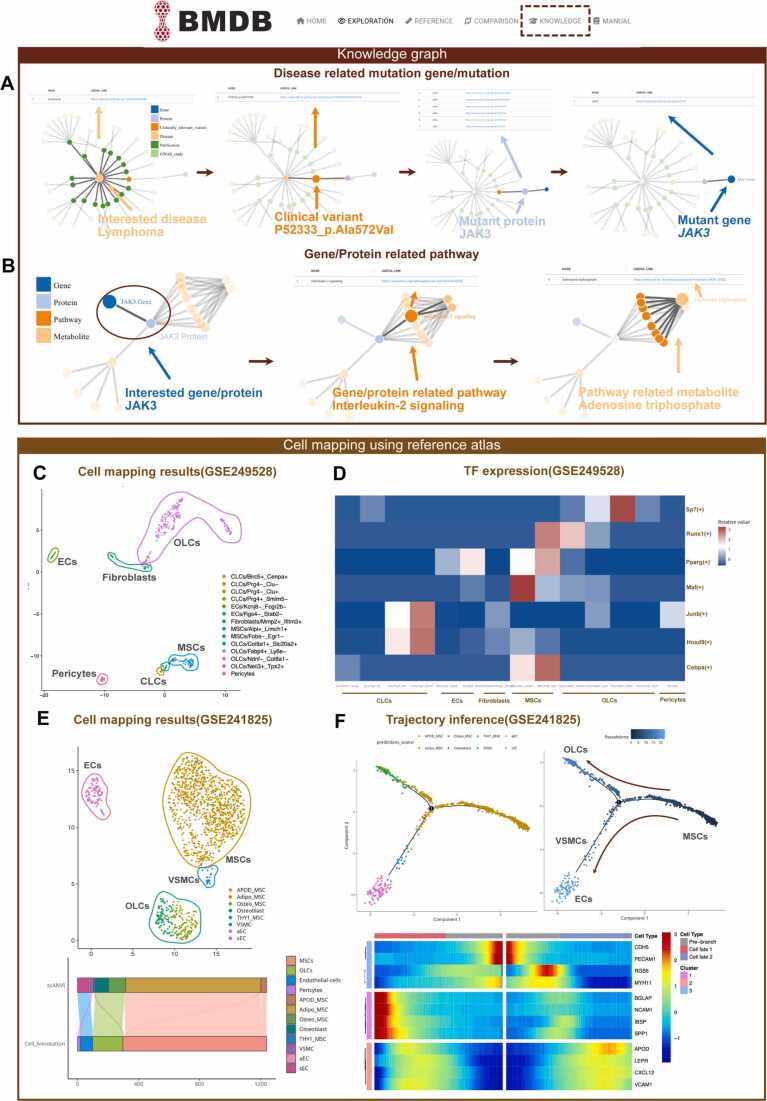
Display of knowledge graph and atlas-based cell mapping for users’ data. (A) A knowledge graph of lymphoma, including associated mutated genes, proteins, publications, and source links. (B) A knowledge graph of JAK3 mutation and its related pathways. (C) UMAP plots presented the cell type and cell lineage annotations of the mouse validation data mapped to the built-in mouse BMN reference using RPCA method. (D) Heatmap showed the differential transcription factors across each cell type within validation data. (E) UMAP plots illustrate the cell type and lineage annotations of the human adult bone marrow dataset following reference mapping to the built-in BMN atlas using scANVI. The Sankey diagram below illustrates the correspondence between the scANVI predicted cell types and the original annotations from the published dataset. (F) The DDRTree trajectories illustrating MSC differentiation toward OLCs and EC/VSMC lineages, visualized by cell-type annotations and pseudotime progression. The lower heatmap shows differentially expressed genes related to branch from branch point 1, which were hierarchically clustered into three groups corresponding to distinct lineage fates.

In the "Protein-related Mutation" page, users could construct a knowledge graph associated with their interested hematologic disease, covering the domains of genes, genetic variants, protein, cell line and publication. In the knowledge graph, nodes represented entities of interest, and edges represented relations between entities. Clicking on a node provides a link to its source information. **(**Fig. 6**A)**. Likewise, on the "Protein-related Pathway" page, users could generate a knowledge graph centered on a protein of interest, focusing on its associated pathways and metabolic processes **(**Fig. 6**B)**. Using the lymphoma knowledge graph as an example, we uncovered a pathogenic mutation in JAK3, which is associated with the IL-2 signaling pathway and affects adenosine triphosphate metabolism.

*De novo analysis for users’ data* BMDB platform incorporates an online de novo analysis function for users' data, enhancing its capabilities as a robust tool for the exploration of bone marrow scRNA-Seq data. As previously stated, the current version of BMDB offers a comprehensive collection of high-quality mouse and human single-cell reference atlases from both adult and fetal stages of the bone marrow niche. The availability of high-quality reference atlases enables the realization of online data analysis for user data in BMDB. Upon uploading the files to the "Cell mapping" page of "Reference" section, cell mapping can be conducted by selecting the appropriate reference atlas and mapping method from the provided drop-down box. Two optional mapping methods, namely RPCA and scANVI, are available for cell mapping.

We first conducted quantitative benchmarking of the reference and reference models embedded in BMDB. Independent mouse (GSE249528) and adult human (GSE241825) bone marrow niche datasets were used for evaluation. The built-in annotation methods of BMDB (scANVI and RPCA) were compared with commonly used tools, including CellTypist, SingleR, and scmap. Accuracy, F1 score, and AUROC were employed as performance metrics. The results showed that scANVI ranked within the top two across all metrics, demonstrating its superior robustness and labeling accuracy compared with other methods (Supplementary Figure 5**A,** Supplementary Table 5). The RPCA-based method also achieved high accuracy and stability. Although its overall performance was not the best, it offered a substantially faster processing speed, particularly advantageous for large-scale datasets. Collectively, the two mapping strategies implemented in BMDB achieve a balance between accuracy and computational efficiency.

In addition, we performed rejection tests using independent PBMC and lung datasets to evaluate out-of-domain (OOD) generalization, while the bone marrow niche was (GSE241825) as the in-domain reference [25], [36]. Confidence score distributions revealed clear separation between in-domain and OOD data, with mean scores of 0.705 for bone marrow niche, 0.586 for PBMC, and 0.437 for lung (Supplementary Figure 5B). As BMDB’s reference framework is trained specifically for bone marrow microenvironment data, annotations remain biologically valid only for non-hematopoietic niche populations. The results demonstrate that both models effectively reject OOD samples (Supplementary Figure 5D). Coverage–threshold and accuracy–threshold curves further showed that prediction stability was maintained up to a confidence threshold of approximately 0.9, with accuracy increasing only at the highest thresholds (≥0.95) accompanied by a sharp loss of coverage (Supplementary Figure 5D). Accordingly, a confidence range of 0.7–0.9 is recommended for users, providing an optimal balance between annotation accuracy and cell coverage.

Subsequently, we utilized two publicly available datasets as examples to demonstrate how BMDB assists users in performing data annotation and downstream analyses [34]. Firstly, a mouse bone marrow niche dataset (GSE249528) was annotated through reference mapping using the RPCA method. The UMAP plot illustrated the outcome of the cell type annotation, including cells of OLCs lineage, CLCs lineage, ECs lineage, Fibroblasts lineage, MSCs lineage, and Pericyte lineage **(**Fig. 6**C)**. To validate the reproducibility of the cell mapping function and the reliability of cell classification, we further characterized the marker genes and highly specific transcription factors for each cell type in the test dataset. All the cellular subsets were easily identified by canonical marker genes, for example, Lepr was found to be highly expressed in MSCs subclusters, while Bglap was highly expressed in OLCs subclusters **(**Supplementary Figure 6**A)**. Moreover, the pySCENIC-based regulon analysis revealed the activation of transcription factors in distinct cell lineages, indicating their lineage-specific regulatory functions. As previously described, Runx1 and Pparg is known for the regulation of the osteogenic differentiation of MSCs cells, while the Cebpa controls the adipogenic differentiation [37], [38]. Sp7 is highly expressed in cells within the OLCs lineage, could inhibit the proliferation of immature osteoblasts, induces osteoblast maturation [39] (Fig. 6D).

Finally, we applied the scANVI method to annotate the healthy subset of the human bone marrow niche dataset (GSE241825). The results were largely consistent with those reported in the original publication, while providing more refined resolution for the endothelial and MSC lineages (Fig. 6**E**, Supplementary Figure 6B). To further explore lineage dynamics, we reconstructed the developmental trajectories using Monocle2, which revealed two principal differentiation branches originating from MSCs. The trajectory topology indicated that MSCs diverged toward OLCs on one branch and EC/VSMCs on the other, reflecting their distinct lineage fates within the bone marrow niche. We then employed Branch Expression Analysis Modeling (BEAM) to identify genes whose expression patterns were regulated in a branch-dependent manner. The resulting heatmap displayed three major gene clusters corresponding to lineage-specific regulatory programs: MSC-related genes (LEPR, CXCL12, VCAM1) predominated in pre-branch states, whereas OLC-associated genes (BGLAP, IBSP, SPP1) and EC-associated genes (CDH5, PECAM1, MYH11) were enriched along the two divergent trajectories (Fig. 6**F**). These coordinated transcriptional changes collectively delineate the bifurcation of MSCs into osteogenic and endothelial lineages, underscoring the regulatory heterogeneity that defines human bone marrow stromal differentiation.

## 4 Discussion

In the physiological state, the regulation of hematopoiesis occurs with precision and speed in order to accommodate the fluctuating needs of the body [40]. The bone marrow HSPC niche assumes a crucial function in this hematopoietic process [41]. This specialized microenvironment primarily consists of mesenchymal stem/stromal cells and vascular endothelial cells [1], [42]. In addition, osteoblasts, chondrocytes, macrophages, and even non-myelinating Schwann cells also contribute to BM HSPC niche [43], [44]. Due to the advancements in scRNA-seq technology, researchers have acquired extensive knowledge regarding the molecular, cellular, and spatial arrangement of the bone marrow (BM) niche [45], [46]. This understanding has been facilitated by the substantial accumulation of scRNA-seq data over the past decade [47], [48], [49]. Nevertheless, the absence of a unified platform that consolidates these data across experiments, samples, and species has limited systematic exploration.

To address this gap, we development BMDB, the first specialized web-based portal for characterizing BM niche cell identities and functions at single-cell resolution. It encompasses the most comprehensive collection of single-cell transcriptomics data pertaining to the BM niche up until the present time. Built upon these data, BMDB offers a unified, web-based platform for data analysis and visualization of the bone marrow niche. Furthermore, the construction and presentation of a comprehensive single-cell reference atlas of the mouse and human fetal bone marrow niche has been accomplished and made available in BMDB. Although several existing atlases include non-hematopoietic components of the bone marrow microenvironment, most contain few non-hematopoietic cells with insufficient lineage resolution, as seen in the bone marrow reference atlases in Azimuth and Tabula Sapiens. Such limitations hinder accurate mapping of users’ datasets and may even lead to misclassification of niche cell populations (Supplementary Table 1**,** Supplementary Figure 6C**-D**). Notably, BMDB distinguishes itself from other databases through its dedicated "KNOWLEDGE" functional module, This feature integrates information on diseases, mutated genes, proteins, biological functions, and metabolic pathways into an interactive network, thereby facilitating comprehensive exploration of the bone marrow microenvironment from phenotypic manifestations to underlying molecular mechanisms across pathological conditions (Supplementary Table 1**)**.

Currently, BMDB has successfully accumulated 36 scRNA-seq datasets pertaining to the BM niche, encompassing 142 normal and 82 pathological samples. We hold the belief that BMDB will serve as a valuable resource for understanding the heterogeneity of the BM microenvironment and the mechanisms of hematopoiesis under both physiological and pathological conditions at single-cell resolution. Despite its comprehensive scope and multi-level integration, BMDB has several limitations. Firstly, most of existing literature on the BM niche derived from animal models, and accordingly 149 of the 224 samples in BMDB are from mouse BM, with the remainder from adult and fetal human BM **(**Supplementary Table 2**)**. Consequently, the reference atlases within the BMDB solely pertain to the mouse and human fetal BM niche. Although the mouse and human datasets encompass data from numerous shared cell types, certain cell types exhibit discernible differences [50]. In addition, the present release primarily integrates single-cell transcriptomic data and does not yet include spatial transcriptomics or other multi-omics modalities such as scATAC-seq or proteomics. Incorporating these data types will deepen analyses of cell–cell interactions, epigenetic regulation, and spatial architecture within the niche, with implementation planned for future versions. Thirdly, although BMDB harmonizes healthy and pathological datasets across species and developmental stages, it currently lacks disease-specific reference atlases. Expanding the resource to include high-quality datasets from distinct hematologic and immune disorders (e.g., leukemia, myelodysplasia, bone marrow fibrosis) will be critical to support disease-oriented analyses and translational applications. Finally, as single-cell datasets are being generated at an accelerating pace, maintaining scalability and automated update strategies remains an ongoing challenge. We are developing a continuous data integration pipeline with standardized quality control and ontology-based annotation to ensure timely incorporation of new datasets and sustainable platform growth. Together, addressing these limitations will allow BMDB to evolve into a more comprehensive and dynamic atlas for exploring the bone marrow microenvironment across health and disease.

In summary, the lack of comprehensive understanding regarding certain crucial components within the human BM, primarily because the investigation of stromal stem/progenitor cells has proven to be challenging [50]. Recent studies are beginning to reveal the complexity and functional diversity of stromal populations, and BMDB is positioned to help synthesize these insights. As the inaugural scRNA-seq database and web tools for the BM niche, BMDB will continue to interpret additional datasets, develop user-friendly analytical modules, and ultimately facilitate further investigations into the HSPC niche within the bone marrow.

## CRediT authorship contribution statement

**Ke Sui:** Writing – review & editing, Writing – original draft, Validation. **Xi Zhang:** Supervision, Resources, Project administration, Funding acquisition. **Chunjing Bian:** Writing – original draft, Methodology, Investigation, Conceptualization. **Yifei Hu:** Investigation, Formal analysis, Data curation. **Jialin Chen:** Writing – original draft, Visualization, Software, Investigation, Formal analysis, Data curation. **Hao Yu:** Visualization, Validation, Software, Methodology, Formal analysis. **Zheng Wang:** Writing – review & editing, Supervision, Resources, Project administration, Funding acquisition, Conceptualization.

## Declaration of Competing Interest

The authors declare that they have no known competing financial interests or personal relationships that could have appeared to influence the work reported in this paper.

## Data Availability

All data including data sources, as well as online website, are freely available at https://bmdb.jflab.ac.cn:8084/ and there is no login requirement. The detail information of all datasets are listed in Supplementary Table 2. The BMDB reference datasets are publicly available at Zenodo (DOI: 10.5281/zenodo.17538576).
