# Supplementary figures and images for: BMDB: An integrated database and web platform for single-cell transcriptomic profiling of bone marrow microenvironment
Source: Comput Struct Biotechnol J. 2025 Nov 15;27:5159–72. doi: 10.1016/j.csbj.2025.11.028 (PMC12666060; doi:10.1016/j.csbj.2025.11.028)

# Supplementary Figure 1

A

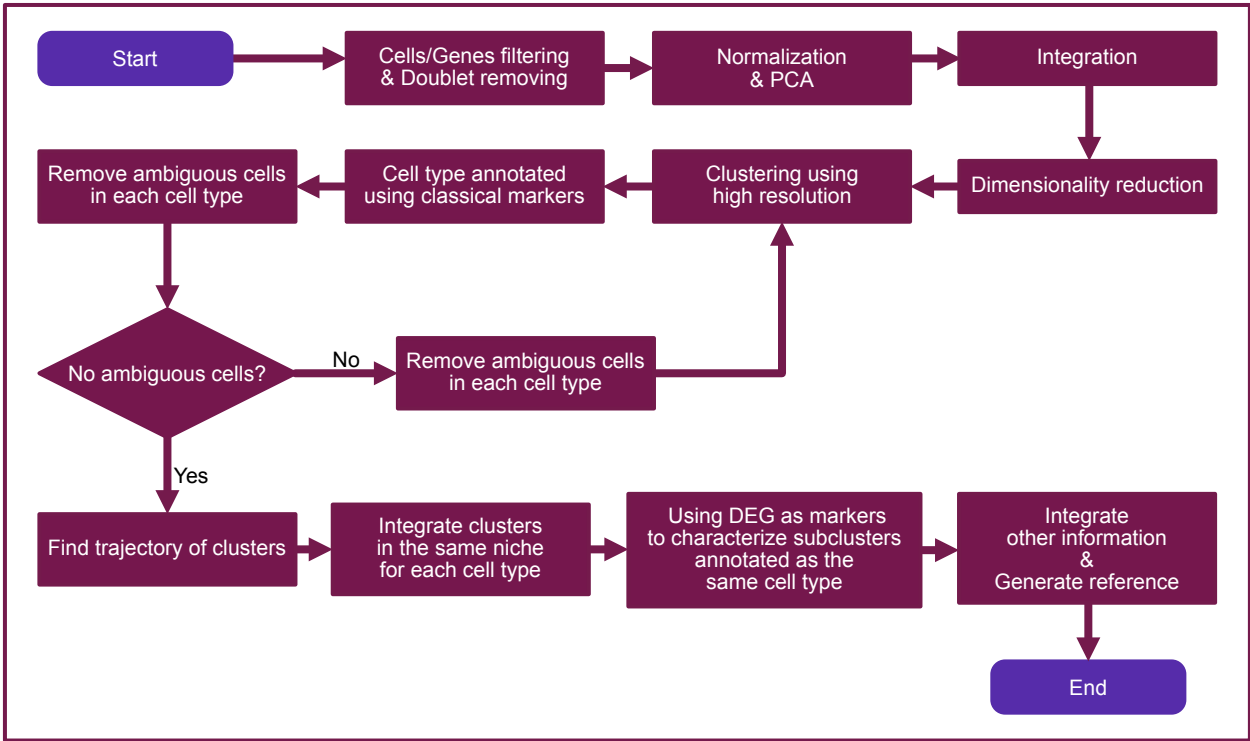

B

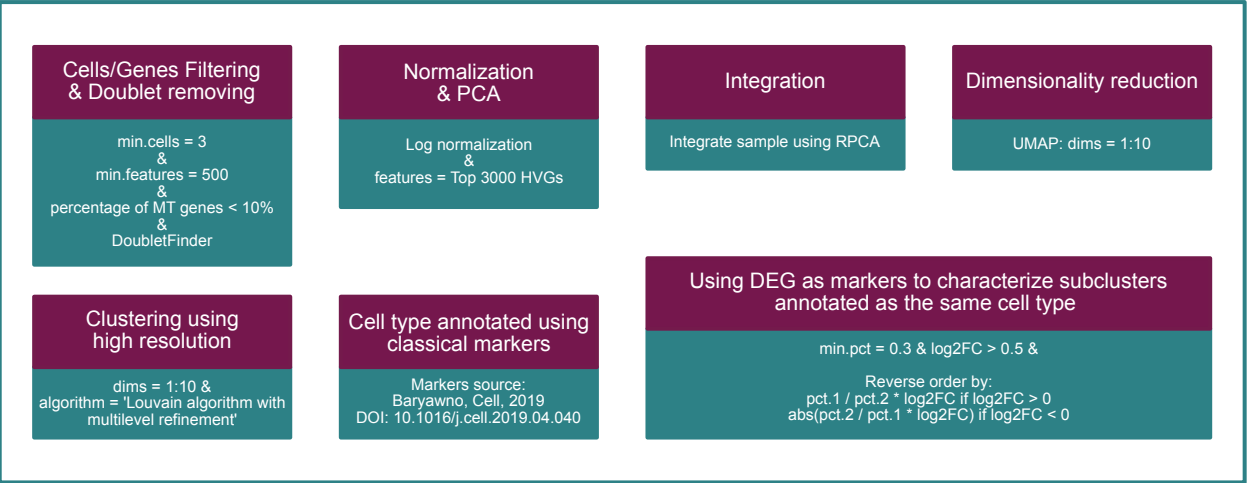

Supplement: Supplementary file 1 — Supplementary material [file mmc1.pdf]

Supplementary Figure 2

Marker gene expression in Human adult BMSC

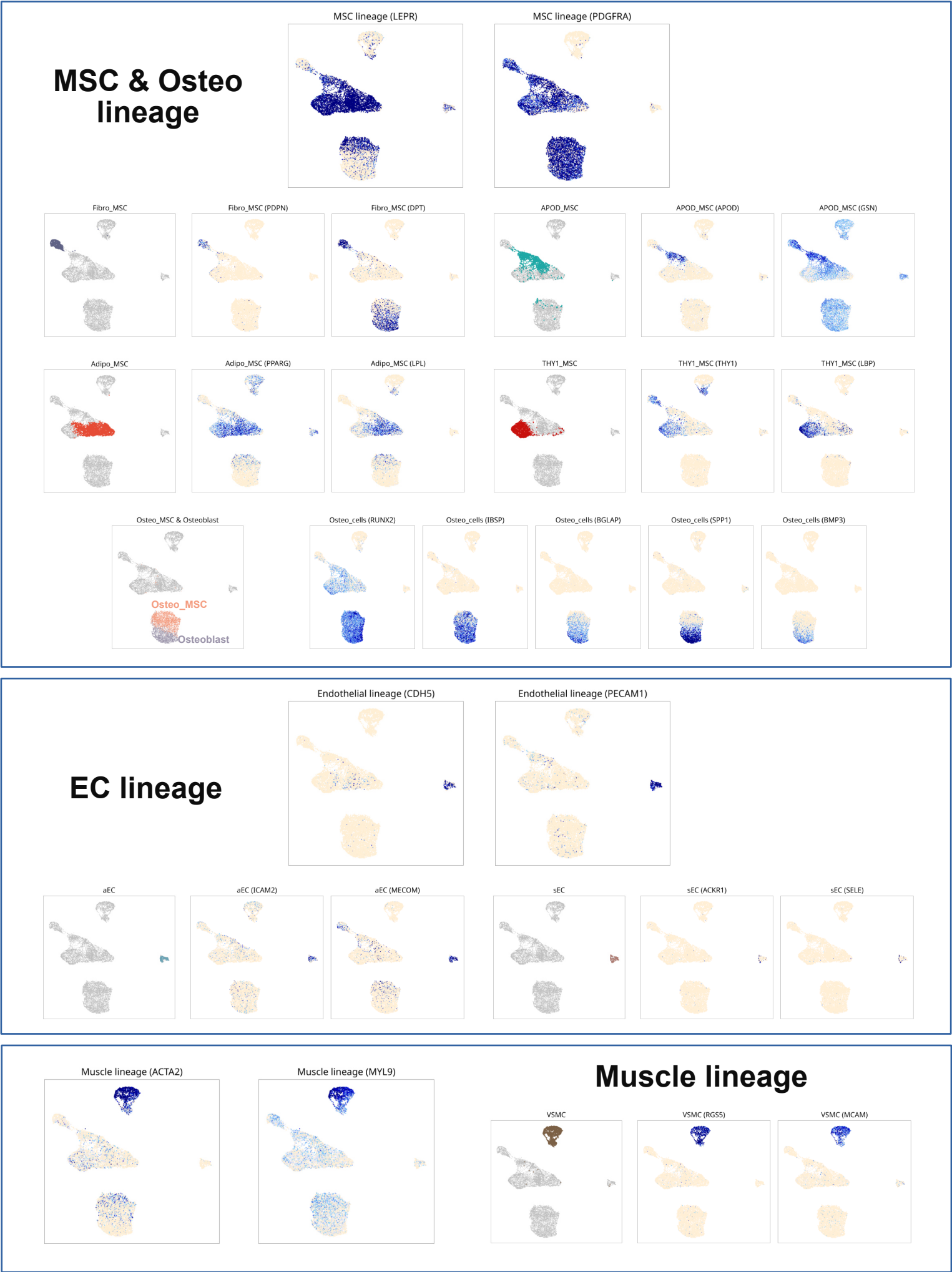

Supplement: Supplementary file 4 — Supplementary material [file mmc2.pdf]

Marker gene expression in Human fetal BMSC

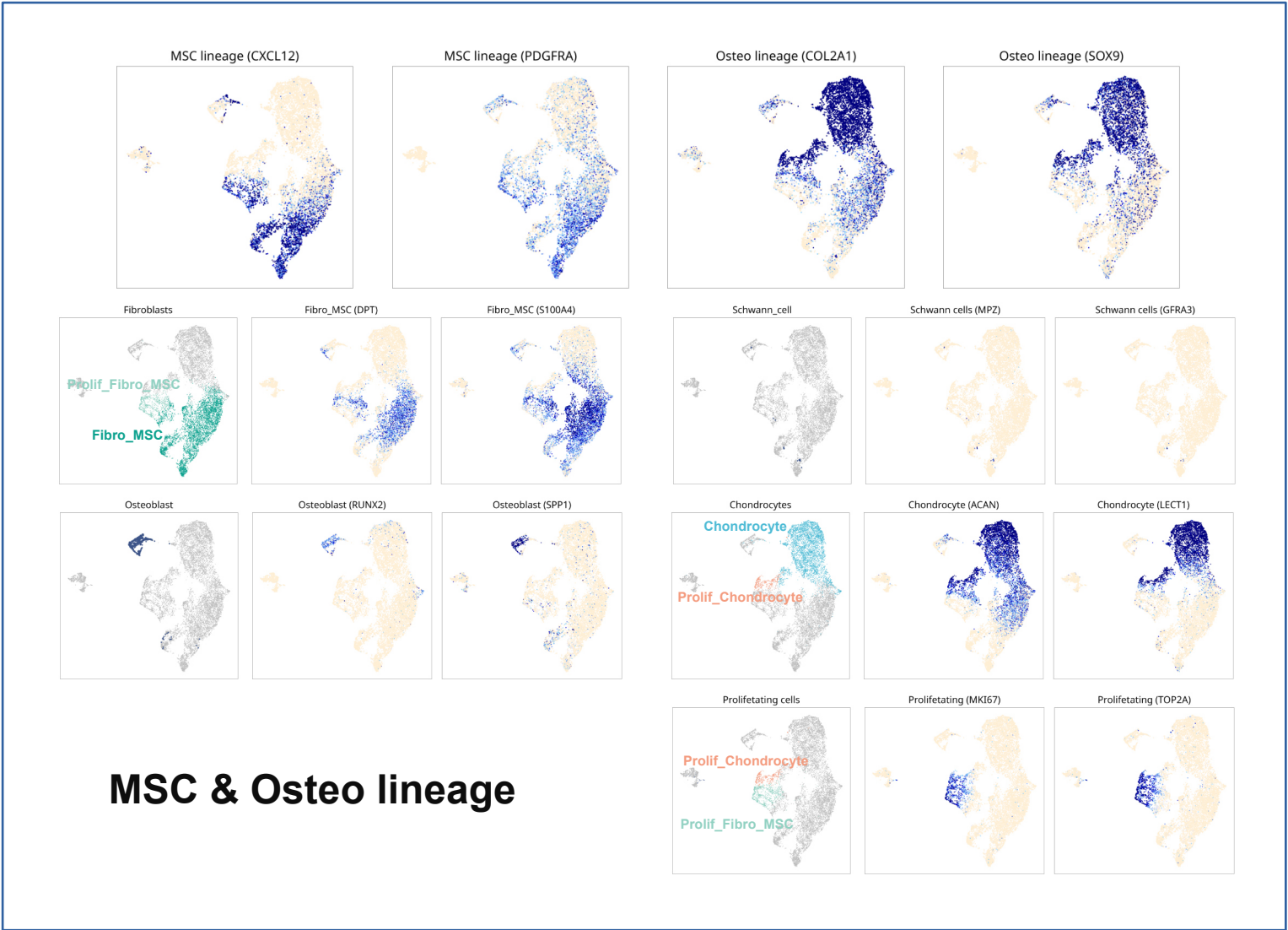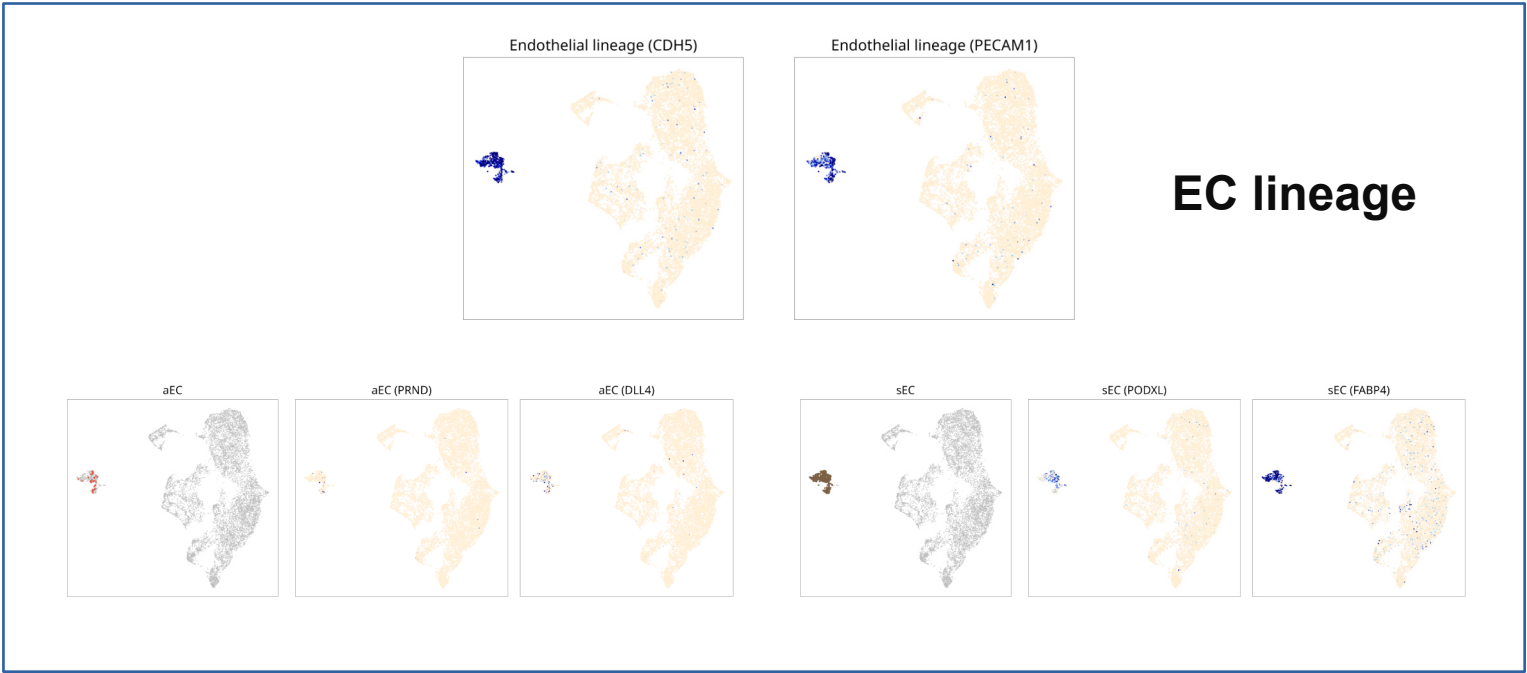

Supplement: Supplementary file 5 — Supplementary material [file mmc3.pdf]

Supplementary Figure 4

Marker gene expression in Mouse BMSC

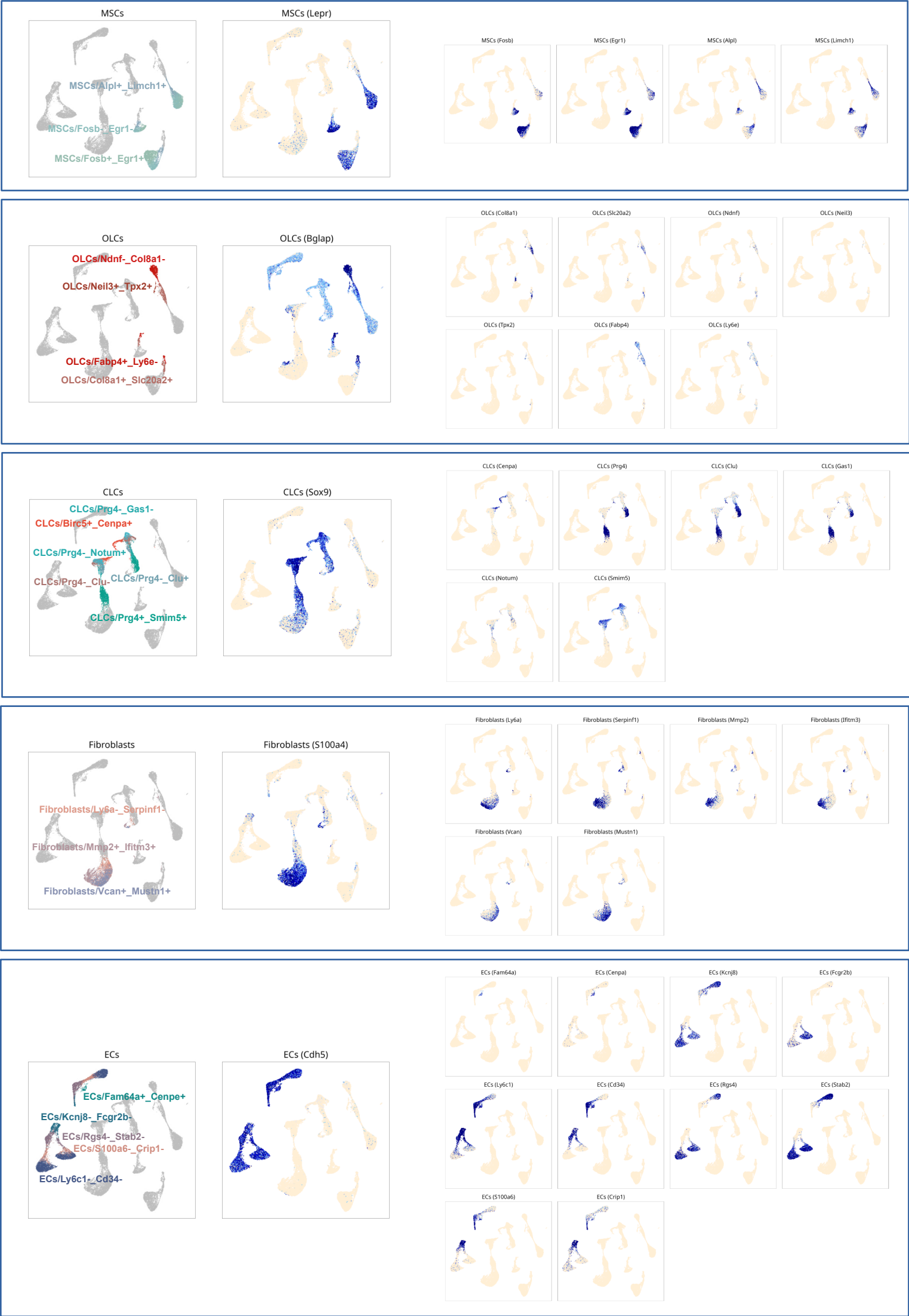

Supplement: Supplementary file 6 — Supplementary material [file mmc4.pdf]

## A Mouse dataset(GSE249528)

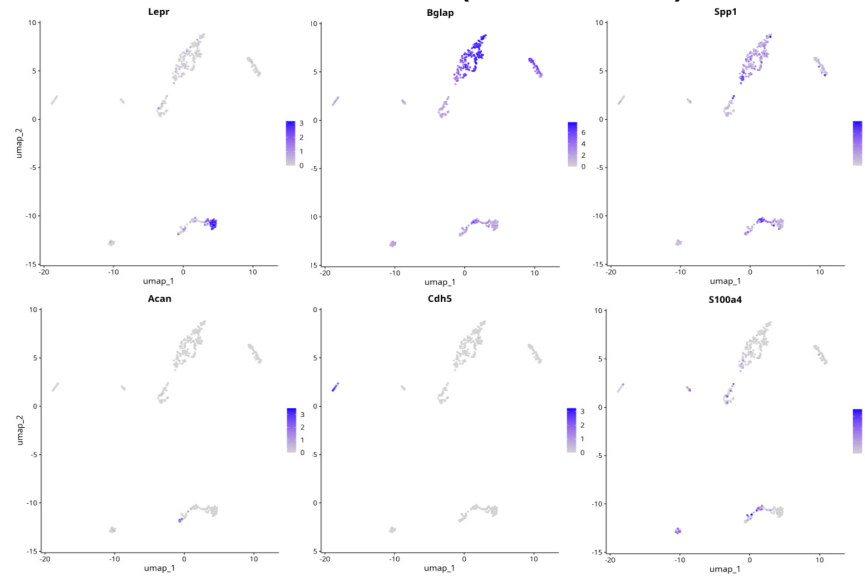

## B Human adult dataset(GSE241825)

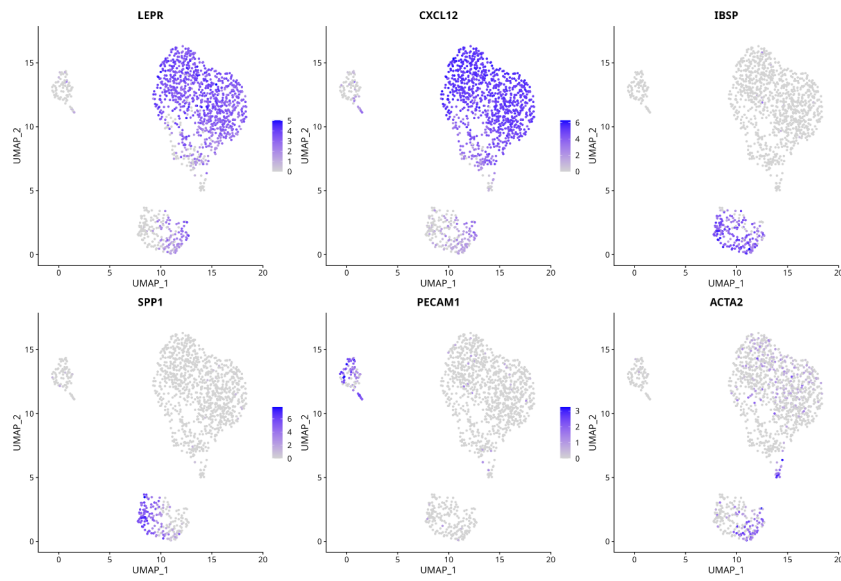

## C

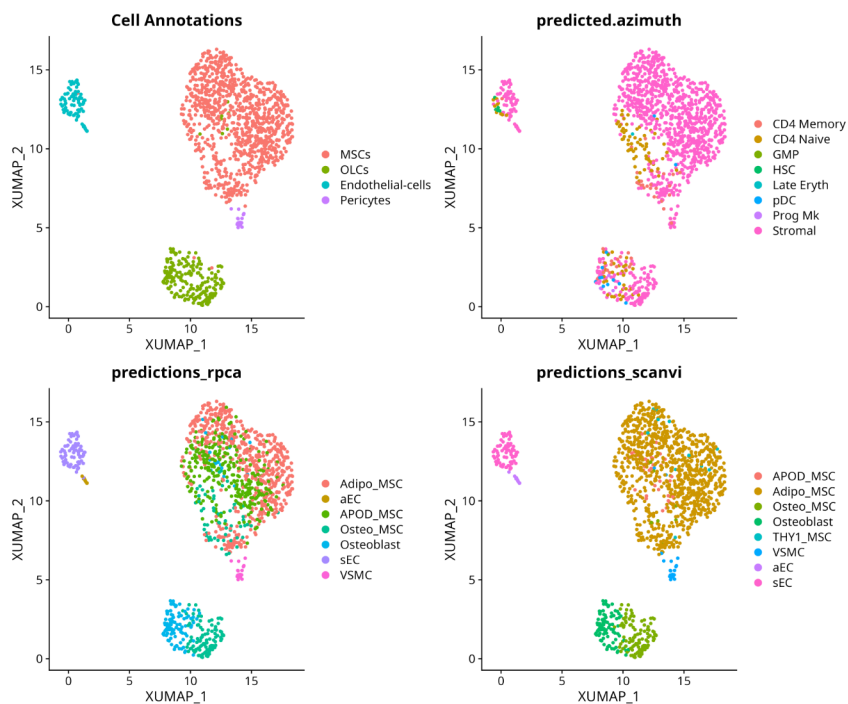

## D

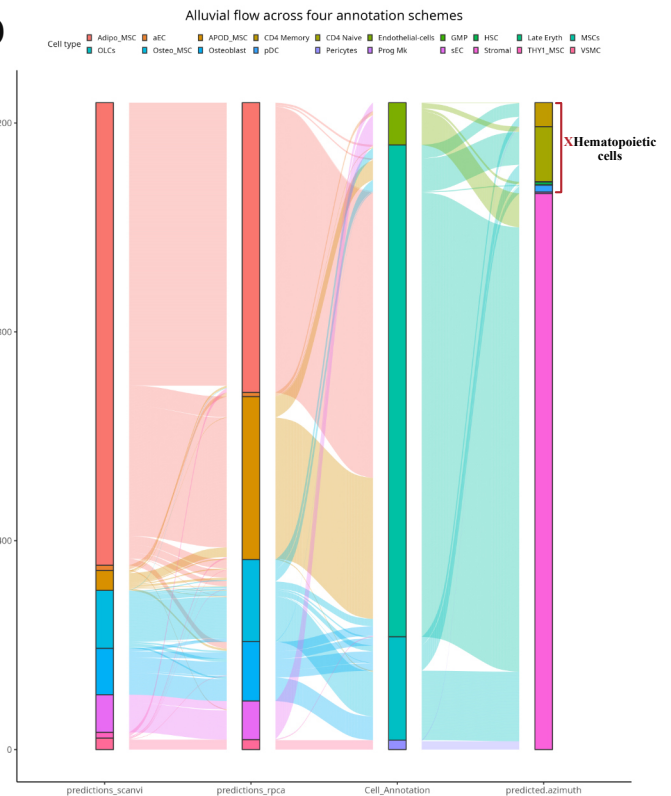

Supplement: Supplementary file 8 — Supplementary material [file mmc6.pdf]
