# Supplementary material for: BMDB: An integrated database and web platform for single-cell transcriptomic profiling of bone marrow microenvironment
Source: Comput Struct Biotechnol J. 2025 Nov 15;27:5159–72. doi: 10.1016/j.csbj.2025.11.028 (PMC12666060; doi:10.1016/j.csbj.2025.11.028)

Supplementary Figure 5

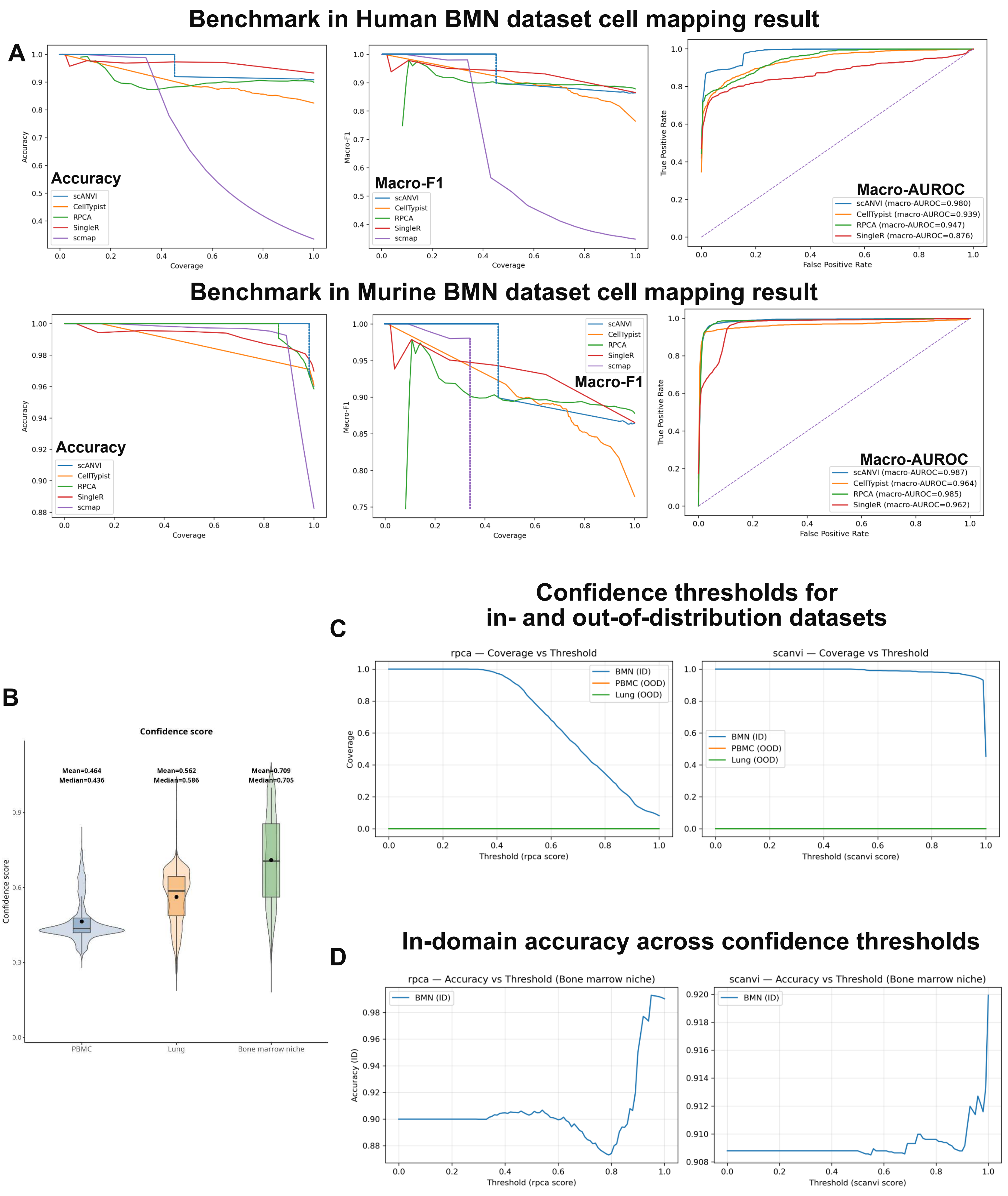

Supplement: Supplementary file 7 — Supplementary material [file mmc5.pdf]
